# Federal Independent Dispute Resolution: A Surgical Problem?

**DOI:** 10.1097/AS9.0000000000000706

**Published:** 2026-09-03

**Authors:** Jacob Guorgui, Lisa M. Knowlton

**Affiliations:** From the * Department of Surgery, Stanford University School of Medicine, Stanford, CA; † Stanford-Surgery Policy Improvement Research and Education Center (S-SPIRE), Palo Alto, CA.

## INTRODUCTION

Recent reports have characterized surgeons as primary contributors to Federal Independent Dispute Resolution (IDR) activity, the arbitration mechanism created to adjudicate out-of-network payment disputes between providers and payers under the 2022 No Surprises Act (NSA).^1,2^ The NSA was intended to mitigate surprise billing by frequently out-of-network clinicians, namely anesthesiologists, emergency medicine physicians, and radiologists.^3,4^ However, prior work has shown that nearly 20% of surgical episodes include out-of-network claims, even when the operating surgeon is in network, raising concern that surgical care may contribute meaningfully to surprise billing.^5^ In turn, the prevalence of surgeons themselves practicing out-of-network, temporal trends in this behavior, and the extent to which this has translated into federal IDR disputes remain unknown. We hypothesize that surgeons play a limited role in federal IDR disputes and out-of-network billing.

## METHODS

Federal IDR data from the Centers for Medicare and Medicaid Services Public Use Files (January 1, 2023 to June 30, 2025) for emergency and nonemergency services were analyzed to determine the share of surgical IDR dispute claims. Claims submitted by surgeons were identified and assigned to subspecialties using a combination of free-text fields and Current Procedural Terminology (CPT) codes to ensure comprehensive capture (Supplemental Appendix https://links.lww.com/AOSO/A668).

Claims data using Merative Marketscan Commercial Database (2014–2023) were additionally analyzed to understand longitudinal trends in rates of out-of-network surgeon inpatient claims in the decade preceding arbitration legislation. Surgical specialties were identified using Marketscan’s provider type variable (STDPROV). Elective cases in which both the surgeon and facility were out-of-network were excluded, as these fall outside the core surprise billing protections of the NSA (Supplemental Appendix, https://links.lww.com/AOSO/A668). Descriptive statistics are reported as counts and percents. All analyses were conducted in R 5.1. Informed consent was waived by the Stanford Institutional Review Board (IRB-40974) for claims data and exempt for the Centers for Medicare and Medicaid Services Public Use Files. STROBE reporting guidelines were followed.

## RESULTS

**FIGURE 1.**
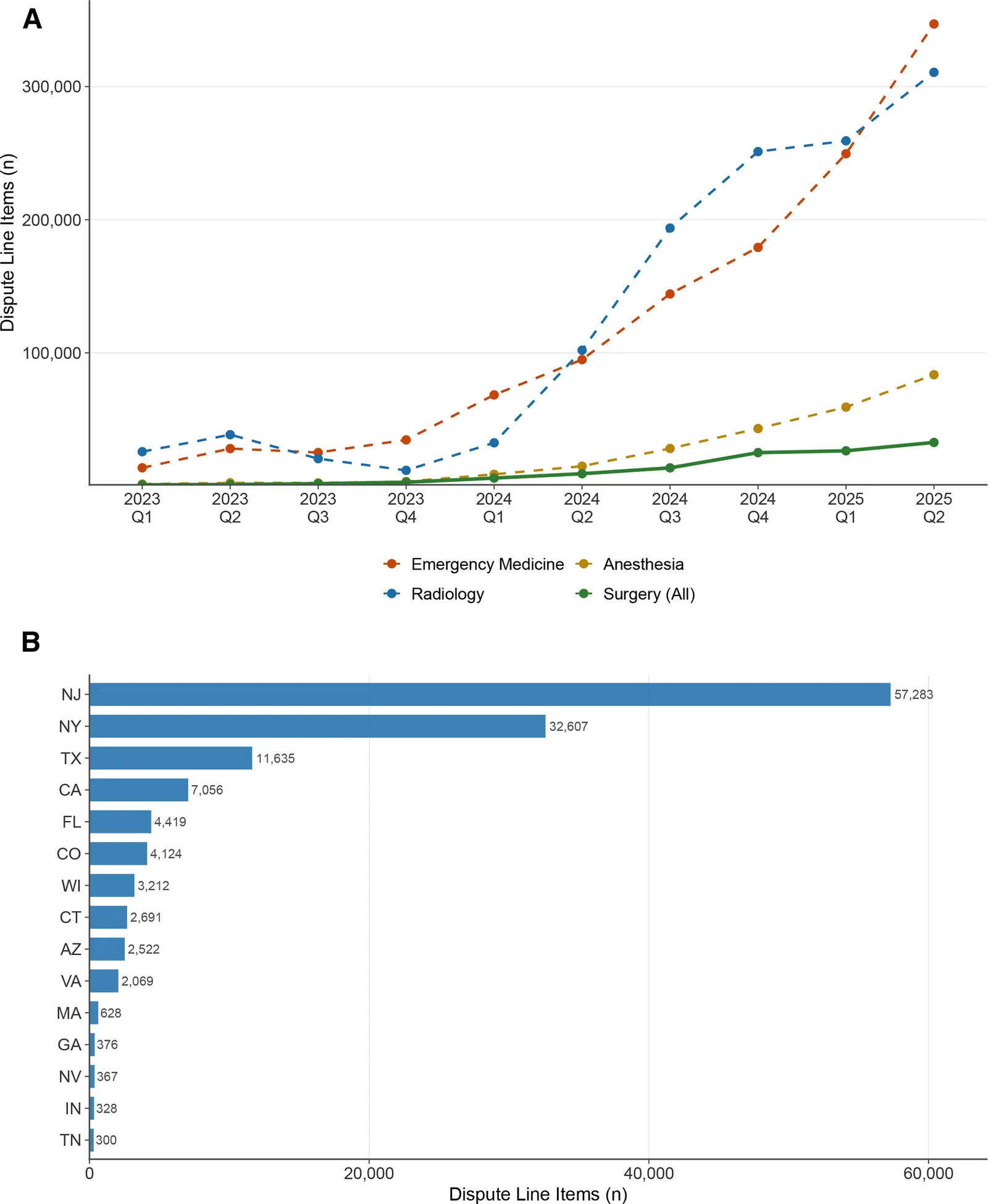
Surgical IDR claims. A, Quarterly number of federal IDR line-item disputes attributable to surgical specialties (all specialty groups combined) compared to emergency medicine, radiology, and anesthesia (dashed lines). B, Total aggregate surgical disputes by state from 2023 Q1 to 2025 Q2.

In total, 5,253,118 disputes were filed during the study period, 131,171 (2.5%) of which were submitted by a surgeon. By quarter, surgeons’ share of all IDR disputes ranged from 1.0% (2023 Q2) to 3.6% (2023 Q4). Specialty distribution was as follows: orthopedic surgery (n = 37,715; 28.8%), plastic surgery (n = 36,347; 27.7%), general surgery (n = 28,896; 22.0%), neurosurgery (n = 24,226; 18.5%), vascular surgery (n = 2784; 2.1%), and cardiothoracic surgery (n = 1203; 0.9%). The overall share of disputes by surgeons over time compared to providers of other specialties is shown in Figure 1A. Claims were heavily concentrated by state (Fig. 1B) and provider; the top 10 NPIs (0.3% of all providers) comprised 20.8% of all surgeon claims. Of note, an additional 23,662 IDR claims were attributable to surgical assistants billing under surgical CPT codes, growing from 20 claims in 2023 Q1 to 7010 in 2025 Q2.

**FIGURE 2.**
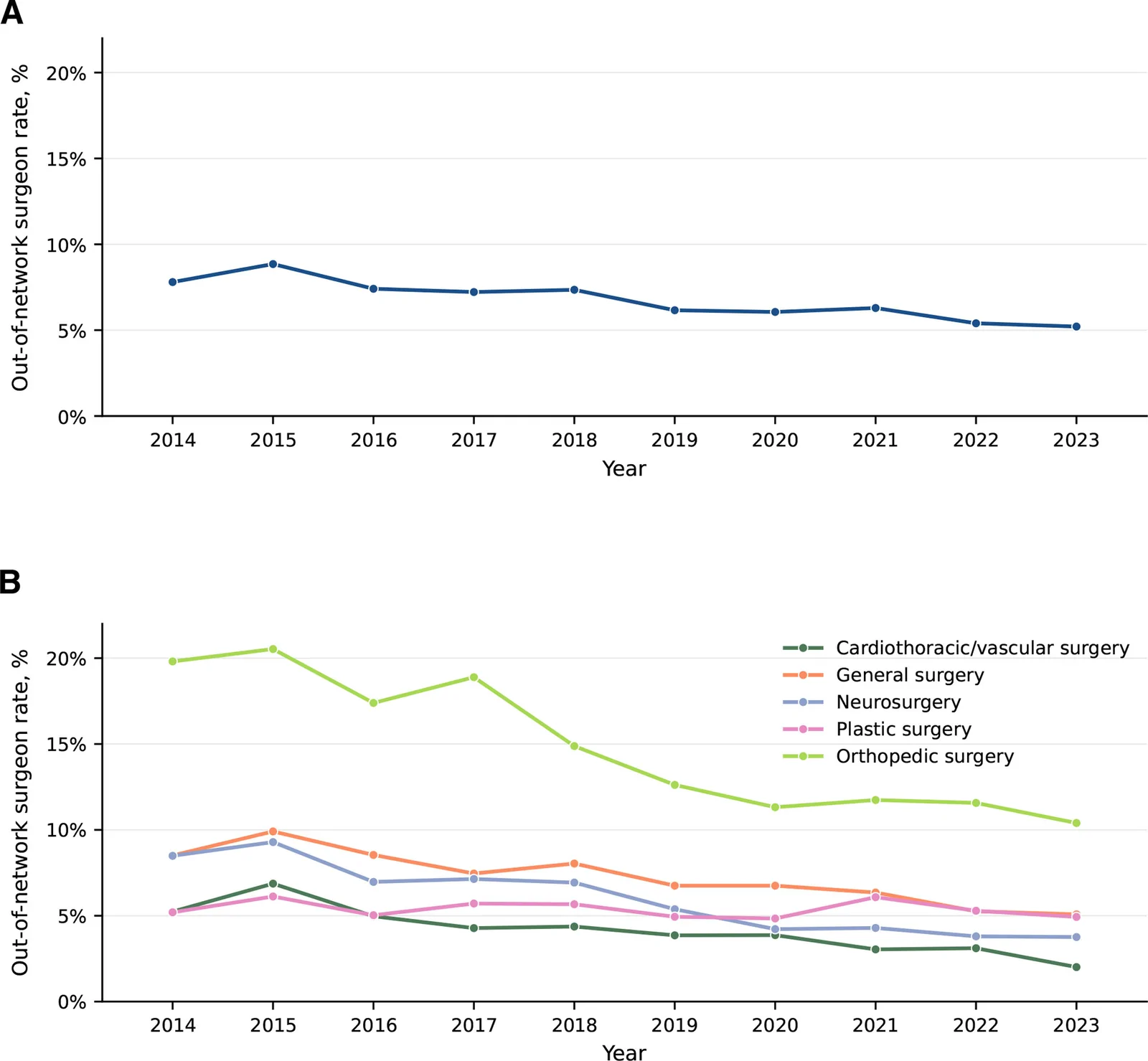
Out-of-network surgeon rate by surgical episode and subspecialty. A, Percentage of all surgical episodes with an out-of-network surgeon between 2013 and 2023. B, Out-of-network surgeon rate by subspecialty. Dashed vertical line in both panels represents passage of the federal NSA in 2022.

Prevailing offers, expressed as a multiple of the qualifying payment amount for each claim, were substantially higher for surgical disputes overall (median 16.5× qualifying payment amount; IQR 5.8–45.2), particularly vascular surgery (39.7×; IQR 13.6–90.1), neurosurgery (29.1×; IQR 7.7–68.1), and cardiothoracic surgery (22.0×; IQR 6.2–81.2), compared with nonsurgical specialties such as radiology (5.3×; IQR 3.2–8.0), anesthesia (3.5×; IQR 2.0–9.2), or emergency medicine (2.9×; IQR 1.9–4.0). Using private-payer claims data, surgeons were out-of-network in 7.0% (88,948/1,267,406) of surgical admissions. The rate of out-of-network surgeons decreased steadily over the decade from as high as 8.9% in 2015 to 5.3% by 2023 (Fig. 2A). Figure 2B shows the trends over time in rates of out-of-network surgeons by surgical specialty, with the highest rates observed among plastic surgeons.

## DISCUSSION

Surgeons were rarely involved in federal IDR disputes, comprising just 2% of claims. Moreover, claims by surgeons appear to be concentrated within a few states and among relatively few providers. These findings are supported by claims data, which revealed a persistent decline in the rates of out-of-network surgeons, even before the federal NSA, suggesting surgeon contracting and market dynamics have evolved to shield patients and payers from surgeon-driven surprise billing. Nonetheless, the high financial stakes of individual surgical claims warrant continued monitoring. Future interventions targeting the federal IDR process should focus on high-volume providers and individual state-level practices, particularly in markets with a history of elevated surprise billing and out-of-network claims (eg, New York and New Jersey). The growing volume of claims submitted by surgical assistants also merits attention.

Limitations include potential undercounting of surgical claims using free-text fields in the PUF files, although 2676 surgical CPT codes were also used to identify additional claims. Subspecialty classification in claims data and by use of CPT code alone in IDR data may also be prone to error. Moreover, MarketScan’s overrepresentation of large employer-sponsored plans may underestimate out-of-network prevalence relative to the broader commercial market. Ultimately, the federal IDR process predominantly involves the specialties the legislation originally aimed to address, while surgeons appear to have a limited role.

## ACKNOWLEDGMENTS

Concept and design: J.G. and L.M.K. Acquisition, analysis, or interpretation of data: J.G. Drafting of the manuscript, critical revision of the manuscript for important intellectual content: J.G. and L.M.K. Statistical analysis: J.G. Administrative, technical, or material support: L.M.K. Supervision: L.M.K.
